# The effect of corticosteroids, antibiotics, and anticoagulants on the development of post-COVID-19 syndrome in COVID-19 hospitalized patients 6 months after discharge: a retrospective follow up study

**DOI:** 10.1007/s10238-023-01153-7

**Published:** 2023-08-08

**Authors:** John Davelaar, Naomi Jessurun, Gerko Schaap, Christina Bode, Harald Vonkeman

**Affiliations:** 1 Department of Rheumatology and Clinical Immunology, Medisch Spectrum Twente, Enschede, The Netherlands; 2 Netherlands Pharmacovigilance Centre Lareb, ‘S-Hertogenbosch, The Netherlands; 3 Faculty of Science and Engineering, University of Groningen, Groningen, The Netherlands; 4 Department of Psychology, Health and Technology, University of Twente, Enschede, The Netherlands

**Keywords:** COVID-19, Post-COVID-19 syndrome, Corticosteroids, Anticoagulants, Antibiotics

## Abstract

To assess the effect of pharmacotherapeutic interventions commonly employed in the management of COVID-19 hospitalized patients on the development of post-COVID-19 syndrome. This study employed two distinct databases, the Medisch Spectrum Twente (MST) clinical database comprising electronic health records of COVID-19 patients hospitalized at MST, and the Post-COVID cohort database which contains follow-up information on the same patients. These databases were integrated to establish the potential relationship between the administration of corticosteroids, antibiotics, or anticoagulants during hospitalization and the occurrence of post-COVID-19 syndrome after a 6-month interval following discharge. A total of 123 patients who were hospitalized due to COVID-19 infection were included in this study. Among these patients, 33 (26.8%) developed post-COVID-19 syndrome which persisted even 6 months after hospital discharge. Multivariate analysis revealed that patients who received treatment with corticosteroids had a significantly lower likelihood (OR 0.32, 95% CI 0.11–0.90) of developing post-COVID-19 syndrome, while no significant association was observed for treatment with antibiotics (OR 1.26, 95% CI 0.47–3.39) or anticoagulants (OR 0.55, 95% CI 0.18–1.71). The findings of this study indicate that corticosteroids exert a significant protective effect against the development of post-COVID-19 syndrome in patients who were hospitalized due to COVID-19 infection. Although a trend towards a protective effect of anticoagulants was observed, it did not reach statistical significance. On the contrary, patients treated with antibiotics were shown to have increased chances of developing post-COVID-19 syndrome, although this effect was also not statistically significant.

**Supplementary Information:**

The online version contains supplementary material available at 10.1007/s10238-023-01153-7.

## Introduction

Severe acute respiratory syndrome (SARS)-coronavirus (CoV)-2 which causes the Coronavirus disease (COVID-19) has taken the world by storm. The impact this virus has had on the global economy, education, travel, and healthcare has been astounding [1–3]. Although physicians initially had difficulty treating this disease, a more or less standardized treatment has been established. Corticosteroids and anticoagulants play an important role in this treatment and target key elements in the disease pathophysiology [4]. As higher incidences of venous thromboembolism (VTE) have been observed for COVID-19 hospitalized patients, varying from 2.6 to 15% for pulmonary embolisms (PE) and 4.6–12% for deep vein thrombosis (DVT), researchers have been prompted to investigate the underlying mechanism [5]. Although this mechanism has not yet been fully elucidated, COVID-19 is thought to cause an exaggerated inflammatory response which in turn leads to endothelial damage and activation of the coagulation cascade. Additionally, micro clotting has been observed in COVID-19 infected patients, hindering oxygen exchange [6]. Furthermore, the immobility of hospitalized patients further increases the risk of thrombotic complications, hence the administration of anticoagulants [7, 8]. Aside from thrombotic complications, COVID-19 patients have been observed with higher levels of inflammatory indices such as C-reactive protein, neutrophils, and interleukins which in turn cause excessive release of pro inflammatory cytokines. These cytokines cause significant damage to the respiratory system leading to pulmonary infections, respiratory failure, and organ damage via immune and inflammatory mediated pathways [9, 10]. Corticosteroids target these inflammatory pathways and have played a vital role in improving clinical outcomes and mortality in patients [11–13]. Another commonly used drug group in the treatment of COVID-19 patients are antibiotics. These drugs are primarily implemented to treat secondary infections and prevent superinfections which has accounted for a significant portion of COVID-19 deaths. That said, some COVID-19 hospitalized patients in severe health states have also been treated empirically with antibiotics, despite the lack of evidence for a beneficial effect [14].

Although healthcare workers have mainly been focused on the treatment of the acute phase, i.e., the viral infection and the associated health symptoms of COVID-19. However it has now become apparent that after the initial infection a certain proportion of patients experience COVID-19 related symptoms for a prolonged period [15]. Due to the lack of a universally accepted definition among the scientific community and the ever-changing availability of information, these post-COVID conditions have been identified by various names. Long haul COVID, chronic COVID, post-acute COVID-19, long COVID, and post-COVID-19 syndrome are a few of the commonly used names. In this study, we have adopted the definitions of COVID conditions as described in the rapid guideline on post-COVID-19 conditions developed in collaboration with the National Institute for Health and Care Excellence (NICE), the Scottish Intercollegiate Guidelines Network (SIGN), and the Royal College of General Practitioners (RCGP). Post-COVID-19 syndrome is defined as “signs and symptoms that develop during or after an infection consistent with COVID-19, present for more than 12 weeks and are not attributable to alternative diagnoses” [16].

As these post-COVID conditions are still in their infancy little is known about the underlying disease pathophysiology and epidemiology. The incidence rate of post-COVID-19 conditions varies widely per study, ranging from more than 30 to 76% after 6 months of symptom onset [17–21]. This variation can be attributed to differences in follow-up length, the definition of the post COVID condition, and the population sample [15].

While much research has been done regarding the safety and efficacy of corticosteroids, antibiotics, and anticoagulants on the treatment of the acute phase of COVID-19 and its concomitant manifestations, little is known regarding the impact these medications have on the development of post-COVID-19 syndrome. No studies were identified looking into a possible association. Given the extent to which these drugs are prescribed, it is therefore imperative to investigate a possible interplay between these drugs and post-COVID-19 syndrome. Identifying the association between pharmacotherapy and the development of post-COVID-19 syndrome allows healthcare providers to make better-informed treatment choices and could contribute to overall patient well-being. Therefore, the aim of this study was to assess the effect of corticosteroids, anticoagulants, and antibiotics on the development of post-COVID-19 syndrome.

## Methods

### Study design

**Fig. 1:**
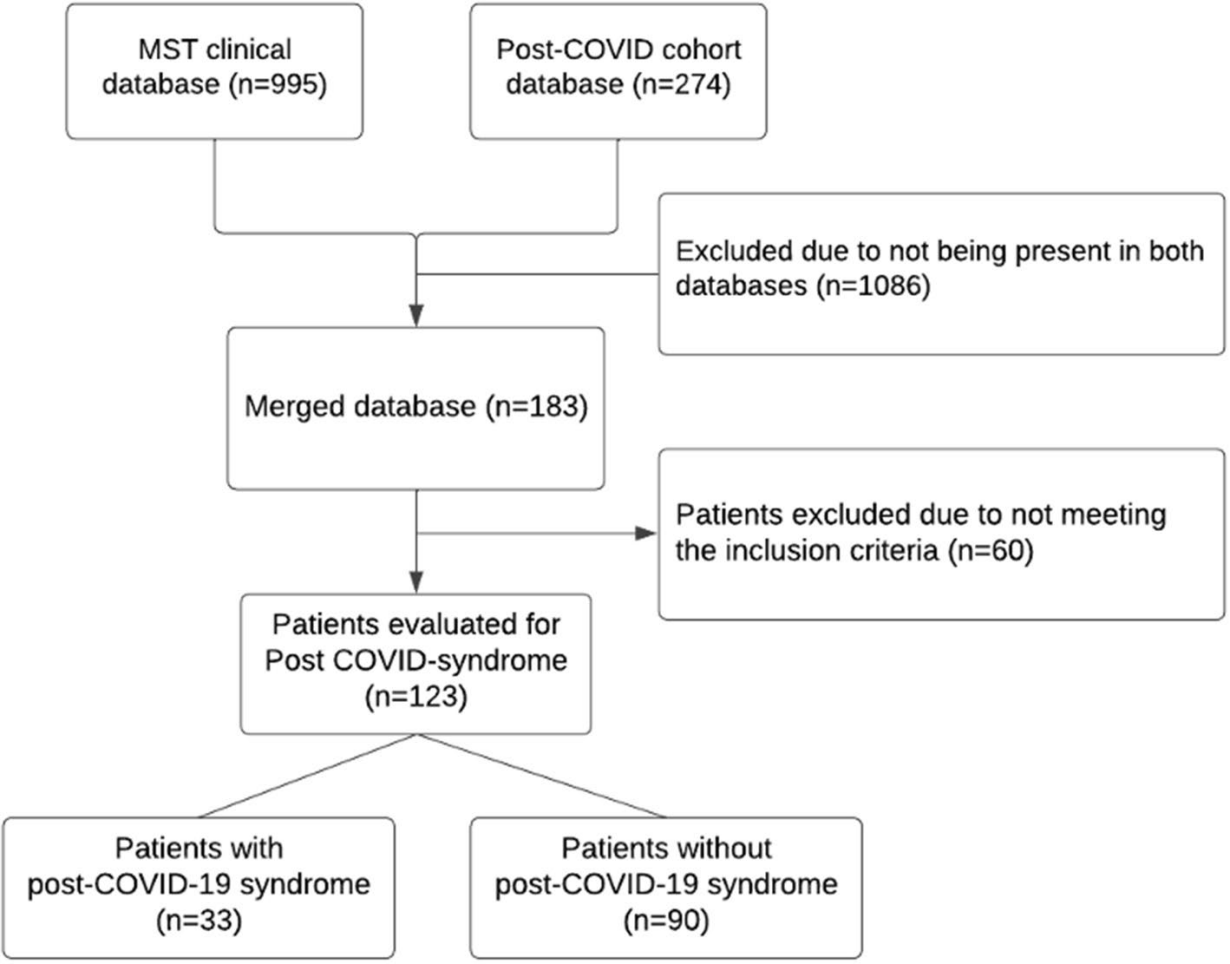
Flowchart of study design

A single-center retrospective follow up study was conducted in which two separate databases; the MST clinical database, which contains electronic health records of COVID-19 hospitalized patients and the Post-COVID cohort database, which contains patient follow-up data, were combined. Using the merged database, it is possible to determine the association between patient pharmacotherapy during the hospital stay and the development of post-COVID-19 syndrome 6 months after hospital discharge (Fig. 1). Three drugs of interest were identified and analyzed, i.e., antibiotics, anticoagulants, and corticosteroids. Separate regression models were made to analyze each individual drug treatment. Drug class effects were measured without taking into account the dose variations or length of use as patients were treated according to the hospital protocol from which physicians rarely deviated.

### Setting

The first database consists of retrospectively sourced patient data (March 2020–September 2021) of 995 COVID-19 hospitalized patients from Medisch Spectrum Twente (MST), Enschede, the Netherlands. Patient data was collected from the time of the first admission with COVID-19 until the last discharge. This database was set up in line with the International Severe Acute Respiratory and Emerging Infection Consortium (ISARIC) rapid COVID-19 case report form (Annex 2) and will hereinafter be referred to as the MST clinical database.

The second database is a separate post-COVID database that consists of 274 patients. This database was originally set up to follow COVID-19 patients’ overall health and well-being after hospital discharge (September 2020—August 2022) using the short form Health survey 36 (SF-36), the EuroQol 5-dimension 5 level descriptive system (EQ-5D-5L), the Medical research council dyspnoe (MRC) survey and the short fatigue survey. Thereby this database will hereinafter be referred to as the Post COVID database.

### Study population

Health questionnaires were sent out to patients quarterly after discharge and focused on physical as well as mental well-being. Demographic information was acquired through a baseline questionnaire and partly through the MST clinical database. For the current study, questionnaires completed 6 months after hospital discharge were coupled via patient study number to the retrospectively collected clinical patient data and selected for analysis. By combining the two databases it is possible to determine which patients develop post-COVID-19 syndrome and the nature of the treatments and medications these patients have undergone while admitted to the hospital. Thus, making it possible to identify possible associations between drug treatments and the development of post-COVID-19 syndrome. The combined database consists of patients 18 years or older that were admitted to the hospital (MST) due to COVID-19 symptoms confirmed using a polymerase chain reaction (PCR) test, who have completed the informed consent form and have sufficient proficiency in the Dutch language.

### Outcomes

**Fig. 2:**
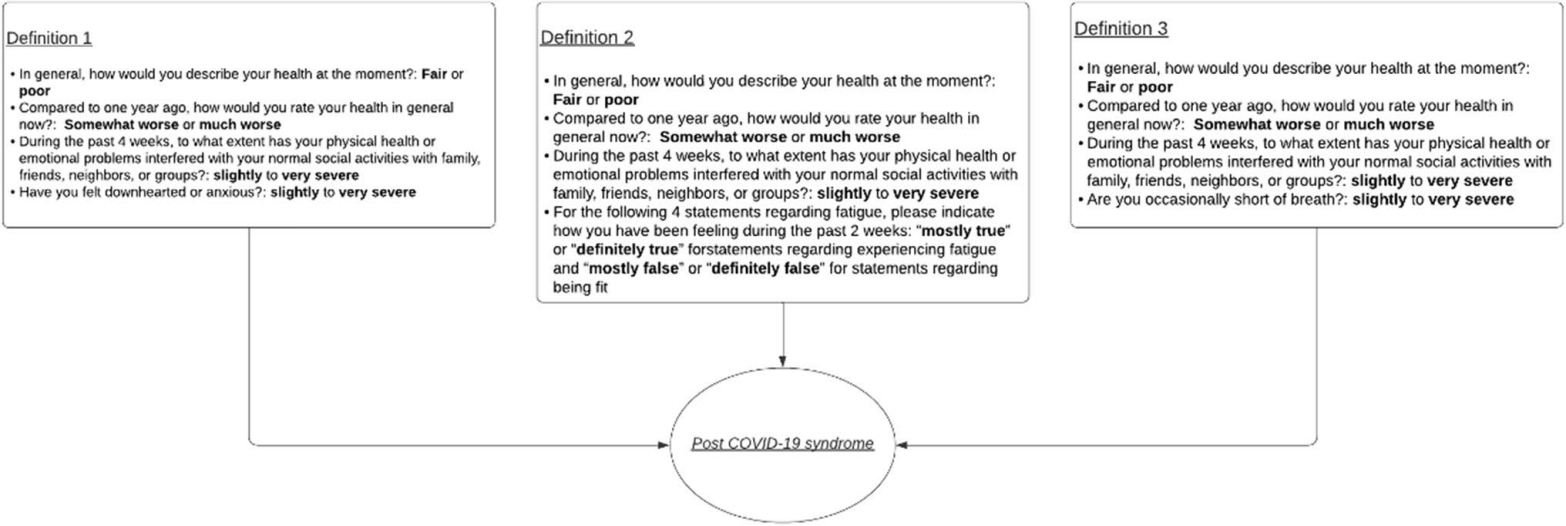
Definitions formulated for developing post-COVID-19 syndrome based on the COVID-19 rapid guidelines

Post-COVID-19 syndrome presence 6 months after hospital discharge was determined using the following questions out of the Post COVID database. Question 1: “In general, how would you describe your health at the moment?”. Question 2: “compared to 1 year ago, how would you rate your health in general now?”. Question 3: “during the past 4 weeks, to what extent has your physical health or emotional problems interfered with your normal social activities with family, friends, neighbors, or groups?”. Question 4: “For the following 4 statements regarding fatigue, please indicate how you have been feeling during the past 2 weeks”. Question 5: “have you felt downhearted or anxious?”. Question 6: “are you occasionally short of breath?”. Using the above-stated questions, three definitions were created to determine post-COVID-19 syndrome status, each consisting of 4 questions (Fig. 2). Definition 1 consists of questions 1, 2, 3 and 5. Definition 2 consist of question 1, 2, 3 and 4. Definition 3 consists of questions 1, 2, 3 and 6. If a patient fit one or more definitions based on the answers they provided, they were categorized as having post-COVID-19 syndrome. A combination of the first question being answered with “fair” or “poor” the second question with “Somewhat worse or much worse now than 1 year ago” and the third question with “slightly to very severe” combined with “mostly true” or “definitely true” for statements regarding experiencing fatigue and “mostly false” or “definitely false” for statements regarding being fit of the fourth question or “slightly to very severe” for the fifth or sixth questions prompted the patient to be categorized as having developed post-COVID-19 syndrome. Due to the broad nature of the definition of post-COVID-syndrome, the aforementioned questions were implemented to encompass the essence and to give body to this definition. The first 3 questions were chosen to capture the burden of post-COVID-19 symptoms and their effect on the physical, mental, and general health of patients. While the fourth, fifth and sixth questions are meant to encompass common symptoms of post-COVID-19 syndrome [22, 23].

### Exposure

The exposure of interest in this study pertained to the use of corticosteroids, antibiotics, or anticoagulants as drug treatments. In accordance with the COVID-19 hospital protocol, patients with a respiratory rate greater than 25 breaths per minute, oxygen requirement of more than 5 L, or a rapidly increasing oxygen requirement were administered 6 mg dexamethasone daily for 10 days (as outlined in Annex 1). In the event that the patient was discharged from the hospital prior to completion of the 10-day period, dexamethasone was prescribed as home medication for the remaining duration. It is noteworthy that a subset of patients were administered alternative corticosteroids, specifically hydrocortisone, methylprednisolone, and prednisolone, due to their existing medical conditions.

Antibiotics were not incorporated into the COVID-19 hospital protocol but were administered preventively or on suspicion of a bacterial infection, ceftriaxone 2000 mg was primarily used. A small group of patients received a different antibiotic (amoxicillin, amoxicillin/clavulanic acid, ceftazidime, ceftriaxone, ciprofloxacin, trimethoprim/sulfamethoxazole, doxycycline, and vancomycin) than ceftriaxone due to treatment for other illnesses.

Hospitalized patients with suspicion or confirmed COVID-19 were administered dalteparin 5000 IE daily, while patients with a bodyweight of 100 kg or more were given 7500 IE daily. Deviation occurred only in cases where the patients had prior usage of specific anticoagulant medications.

### Variables

The association model included five covariates: gender, age, body mass index (BMI), COVID severity, and comorbidity. Age was grouped into more or less equal group sizes to be able to identify trends in the data while BMI was grouped according to the World Health Organization (WHO) classification [24]. COVID severity was determined using the National Early Warning Score 2 (NEWS 2) at hospital admission and could be categorized as low, low-medium, medium, and high [25]. Due to the relatively small sample size of the dataset, comorbidities were coded into the broadest parent term according to the International Statistical Classification of Diseases and Related Health Problems (ICD-11) after which the comorbidities were grouped into having a direct or indirect influence on COVID-19, based on literature. This was done to better capture the effect of the variable comorbidity by increasing the effect size of said variable. Diseases of the circulatory system, the respiratory system, the immune system, and endocrine, nutritional, or metabolic diseases were considered as having a potential direct influence on COVID-19. While diseases of the blood or blood-forming organs, the genitourinary system, the musculoskeletal system or connective tissue, the nervous system, neoplasms, sleep–wake disorders, factors influencing health status or contact with health services and mental, behavioral, or neurodevelopmental disorders were considered as having a potential indirect influence on COVID-19 [26, 27].

### Data analysis

To identify potential differences between the distributions of the COVID-19 drug treatment in non-post-COVID-19 syndrome and post-COVID-19 syndrome patients, Pearson’s chi-squared test was used in combination with relative risks to measure the effect size in the univariate analysis. Furthermore, using a binomial logistic regression, the association between corticosteroids, antibiotics, or anticoagulants and the development of the post-COVID-19 syndrome was determined. A p-value lower than 0.05 was considered to be significant. Missing data was imputed using the expectation maximization method. A missing value of 5% or lower was considered to be acceptable for imputation. Furthermore, data imputation was only necessary in one case. Statistics were performed in R (version 4.0.3) and SPSS (version 28.0.1.0).

### Patient and public involvement

Patients and/or the public were not involved in the design, or conduct, or reporting, or dissemination plans of this research.

## Results

A total of 123 patients met the inclusion criteria of which, 33 patients (26.8%) had developed and were still affected by post-COVID-19 syndrome 6 months after hospital discharge (Table 1). The univariate analysis shows no statistically significant difference between the use of corticosteroids for patients who have and have not developed post-COVID-19 syndrome (*p* = 0.14). The same results can be seen for patients treated with anticoagulants (*p* = 0.34) or antibiotics (*p* = 0.96) (Table 2).

**Table 1:** Characteristics of study population

	Total	Post-COVID-19 syndrome	Non-post-COVID-19 syndrome
Patients (%)	n = 123	n = 33	n = 90
Gender (female) (%)	47 (38.2%)	17 (51.5%)	30 (33.3%)
Age (years), mean ± SD	62.1 ± 9.5	58.5 ± 10.6	63.4 ± 8.8
Comorbidity (%)			
Direct influence on COVID-19^a^	85 (69.1%)	20 (60.6%)	65 (72.2%)
COVID severity^b^ (%)			
Low	53 (43.1%)	14 (42.4%)	39 (43.3%)
Low-medium	8 (6.5%)	0 (0.0%)	8 (8.9%)
Medium	24 (19.5%)	6 (18.2%)	18 (20.0%)
High	38 (30.9%)	13 (39.4%)	25 (27.8%)
Drug treatment			
Antibiotics	89 (72.4%)	24 (72.7%)	65 (72.2%)
Anticoagulants	100 (81.3%)	25 (75.8%)	75 (83.3%)
Prophylatic^c^	94 (94.0%)	24 (96.0%)	70 (93.3%)
Corticosteroids	73 (59.3%)	16 (48.5%)	57 (63.3%)
Antibiotic + Anticoagulant	71 (57.7%)	18 (54.5%)	53 (58.9%)
Antibiotic + Corticosteroids	53 (43.1%)	13 (39.4%)	40 (44.4%)
Anticoagulant + Corticosteroid	64 (52.0%)	13 (39.4%)	51 (56.7%)
Antibiotic + Anticoagulant + corticosteroid	46 (37.4%)	11 (33.3%)	35 (38.9%)
Hospital stay duration (days), mean ± SD	12.5 ± 11.3	10.2 ± 8.6	13.3 ± 12.1

^a^Diseases of the circulatory system, the respiratory system, the immune system, and endocrine, nutritional, or metabolic diseases

^b^Determined using the NEWS-2 score

^c^Remaining anticoagulant therapy was therapeutically dosed

**Table 2:** Multivariate analysis results specified per drug treatment

Drug treatment	Odds ratio	95% confidence interval (CI)
Antibiotics	1.26	0.47–3.39
Anticoagulants	0.55	0.18–1.71
Corticosteroids	0.32	0.11–0.90
Antibiotic + Anticoagulant	0.81	0.35–2.27
Antibiotic + Corticosteroids	0.79	0.31–1.99
Anticoagulant + Corticosteroid	0.337	0.12–0.93
Antibiotic + Anticoagulant + corticosteroid	0.78	0.29–2.06

The univariate analysis showed no significant difference between the individual exposure groups (corticosteroids, antibiotics, or anticoagulants) and development of post-COVID-19 syndrome. While the multivariate analysis showed that corticosteroid treatment in patients hospitalized for COVID-19 was associated with a significantly smaller chance (OR 0.32, 95% CI 0.11–0.90, *p* = 0.03) of developing post-COVID-19 syndrome. Anticoagulant treatment in patients hospitalized for COVID-19 was associated with a smaller chance (OR 0.55, 95% CI 0.18–1.71, *p* = 0.30) of developing post-COVID-19 syndrome although this effect was not found to be significant. While antibiotic treatment in patients hospitalized for COVID-19 was associated with a greater chance (OR 1.26, 95% CI 0.47–3.39, *p* = 0.65) of developing post-COVID-19 syndrome compared to patients not treated with antibiotics, it was not statistically significant. Combinations of drug treatments showed no significant changes in lessening the chance of developing post-COVID-19 syndrome.

## Discussion

Patients treated with corticosteroids were associated with significantly lower incidences of post-COVID-19 syndrome. While patients treated with anticoagulants were also observed benefitting from a protective effect from developing post-COVID-19 syndrome, this effect was not significant. Antibiotic treatment on the other hand seemed to increase incidences of post-COVID-19 syndrome, although not significantly.

Although information regarding post-COVID-19 syndrome disease pathophysiology is scarce, corticosteroids’ effect on this disease may be explained by the hyperactivation of (chronic) inflammatory and immunological pathways post-COVID-19 syndrome induces and the anti-inflammatory and immunomodulating mechanism of action of corticosteroids. Among others, Corticosteroids decrease the production of pro-inflammatory cytokines and chemokines, stimulate the release of anti-inflammatory cytokines, and suppress lymphocyte activation and production of immunoglobulins, which play important roles in the disease progression [15, 28].

Patients infected with COVID-19 have been observed developing micro clots which in turn obstruct capillaries, hinder oxygen exchange, and could cause post-COVID-19 related symptoms. This in turn may be a potential explanation for the seemingly protective effect against post-COVID-19 syndrome of anticoagulants [6]. In contrast to corticosteroids and anticoagulants, antibiotics seemed to increase the chances of developing post-COVID-19 syndrome. As antibiotics are not part of Medisch Spectrum Twente’s COVID-19 treatment protocol, we can infer that patients who had received antibiotics were primarily treated for secondary infections which in turn suggests a graver patient health state. This is in line with our regression model which also shows a possible trend for higher chance of developing post-COVID-19 syndrome with increasing disease severity. However, this finding was not significant. Furthermore, some studies have better identified the role of the gut microbiome in COVID-19 patients, which show that antibiotics cause a dysbiosis in the gut microbiome which is closely involved in immune system regulation through the gut–lung axis and gut-brain axis amongst others. An imbalance can lead to more pro-inflammatory mediators and immune cell activation which in turn perpetuates the disease [29–32].

Besides the underlying disease mechanisms, the significance of these results may be explained by the discriminatory ability of the aforementioned drugs. When patients are admitted to MST due to COVID-19, anticoagulants are administered as per hospital protocol, whereas corticosteroids are only administered when certain patient criteria are met. These criteria, such as respiratory rate and oxygen requirement are directly linked to disease activity and therefore have a higher discriminatory ability.

Seeing as post-COVID-19 conditions had only just started to emerge at the commencement of this study, no treatment or predisposing factors for these conditions were known. We can therefore infer that the COVID-19 hospitalized patients were treated as per hospital protocol and thus received similar medical treatment, explaining why no differences were found in administered drug treatment among patients.

The current study has several limitations. Firstly, the included sample size was limited which has direct implications on the power of our study. Secondly, drug class effects were measured for each drug group without taking into account possible dose variations or treatment duration as patients were treated as per hospital protocol. Thirdly, comorbidities were grouped into having a direct or indirect influence on COVID-19. Due to the relatively small sample size, it was necessary to include limited disease groups so as to not skew the data. For this reason, based on literature review and expert opinion it was decided to only include a select few disease groups as these were considered to be the most impactful on COVID-19 severity.

This study provides more insight into the effect of commonly used drug treatments for COVID-19 hospitalized patients on the development of post-COVID-19 syndrome. Although corticosteroids are primarily used for the treatment of COVID-19 hospitalized patients fitting certain respiratory criteria, this study shows corticosteroids reducing the probability of developing post-COVID-19 syndrome related symptoms after the acute infection. The observed protective effect is a new and unexpected finding which may have implications for daily practice in healthcare in relation to post COVID-19 syndrome prevention. Although the results indicate the added value of using corticosteroids for the prevention of post-COVID-19 syndrome, it is equally important not to overuse these drugs given the immunosuppressive effects and the development of infections. For this reason, more studies are needed to not only substantiate these findings but also to find an optimal treatment dose and length for corticosteroid use.

## Conclusion

COVID-19 hospitalized patients in MST underwent the same treatments as per hospital protocol. No distinction was made between patients with predisposing factors for post-COVID-19 syndrome. Corticosteroids have shown a significant protective effect on the development of post-COVID-19 syndrome in this study. Further research is required to validate these findings and to yield improved prevention and management strategies for post-COVID-19 syndrome.

### Supplementary Information

Below is the link to the electronic supplementary material.

Supplementary file1 (PDF 932 kb)

## Data Availability

The datasets generated and analyzed for the current study are not publicly available due to legal restrictions related to data privacy protection but are available from the corresponding author on reasonable request.
