# Correction to: The effect of corticosteroids, antibiotics, and anticoagulants on the development of post-COVID-19 syndrome in COVID-19 hospitalized patients 6 months after discharge: a retrospective follow up study

**DOI:** 10.1007/s10238-023-01217-8

**Published:** 2023-10-30

**Authors:** John Davelaar, Naomi Jessurun, Gerko Schaap, Christina Bode, Harald Vonkeman

**Affiliations:** 1 Department of Rheumatology and Clinical Immunology, Medisch Spectrum Twente, Enschede, The Netherlands; 2 Netherlands Pharmacovigilance Centre Lareb, ‘S-Hertogenbosch, The Netherlands; 3 Faculty of Science and Engineering, University of Groningen, Groningen, The Netherlands; 4 Department of Psychology, Health and Technology, University of Twente, Enschede, The Netherlands

**Correction to: Clinical and Experimental Medicine** https://doi.org/10.1007/s10238-023-01153-7

The original version of this article unfortunately contained a mistake. The corrected details are provided below:

In abstract, first sentence, which previously read:

To assess the effect of pharmacotherapeutic interventions commonly employed in the management of COVID-19 hospitalized patients on the development of post-COVID-19 syndrome.

Should read:

This assessment evaluates the effect of pharmacotherapeutic interventions commonly employed in the management of COVID-19 hospitalized patients on the development of post-COVID-19 syndrome.

