# Supplementary material for: The effect of corticosteroids, antibiotics, and anticoagulants on the development of post-COVID-19 syndrome in COVID-19 hospitalized patients 6 months after discharge: a retrospective follow up study
Source: Clin Exp Med. 2023 Aug 8;23(8):4881–8. doi: 10.1007/s10238-023-01153-7 (PMC10725368; doi:10.1007/s10238-023-01153-7)
Supplement: Supplementary file 1 — Supplementary file1 (PDF 932 kb) [file 10238_2023_1153_MOESM1_ESM.pdf]

Annex 1 – MST COVID-19 treatment protocol

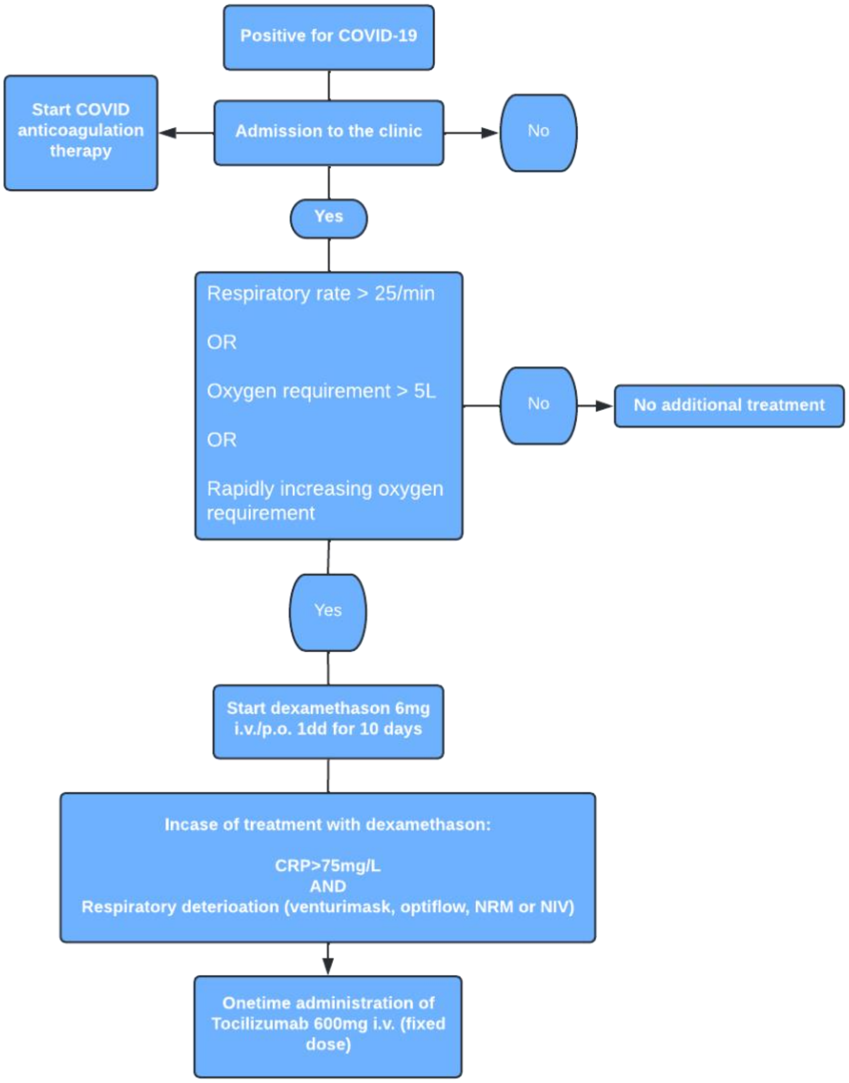

Annex 2 – ISARIC rapid case record form

Global COVID-19 Clinical Platform

NOVEL CORONAVIRUS (COVID-19) - RAPID VERSION

DESIGN OF THIS CASE RECORD FORM (CRF)

This CRF has 3 modules:

Module 1 to be completed on the first day of admission to the health centre.

Module 2 to be completed on first day of admission to ICU or high dependency unit. Module 2 should also be completed daily for as many days as resources allow. Continue to follow-up patients who transfer between wards.

Module 3 to be completed at discharge or death.

GENERAL GUIDANCE

- The CRF is designed to collect data obtained through examination, interview and review of hospital notes. Data may be collected retrospectively if the patient is enrolled after the admission date.
- Participant Identification Numbers consist of a site code and a participant number. You can obtain a site code and register on the data management system by contacting ncov@isaric.org. Participant numbers should be assigned sequentially for each site beginning with 0001. In the case of a single site recruiting participants on different wards, or where it is otherwise difficult to assign sequential numbers, you can assign numbers in blocks or incorporate alpha characters. E.g. Ward X will assign numbers from 0001 or A001 onwards and Ward Y will assign numbers from 5001 or B001 onwards. Enter the Participant Identification Number at the top of every page.
- Data are entered to the central electronic REDCap database at https://ncov.medsci.ox.ac.uk or to your site/network's independent database. Printed paper CRFs may be used and the data can be typed into the electronic database afterwards.
- Complete every section. Questions marked "If yes,..." should be left blank when they do not apply (i.e. when the answer is not yes).
- Selections with square boxes (☐) are single selection answers (choose one answer only).
- Selections with circular boxes (O) are multiple selection answers (choose all that apply).
- Mark 'Unknown' for any data that are not available or unknown.
- Avoid recording data outside of the dedicated areas.
- If using paper CRFs, we recommend writing clearly in ink, using BLOCK-CAPITAL LETTERS.
- Place an (X) in the boxes to mark the answer. To make corrections, strike through (-------) the data you wish to delete and write the correct data above it. Please initial and date all corrections.
- Please keep all of the sheets for a single participant together e.g. with a staple or participant-unique folder.
- Please transfer all paper CRF data to the electronic database. All paper CRFs can be stored by the institution responsible for them. All data should be transferred to the secure electronic database.
- Please enter data on the electronic data capture system at https://ncov.medsci.ox.ac.uk. If your site would like to collect data independently, we can support the establishment of locally hosted databases.
- Please contact us at ncov@isaric.org. If we can help with databases, if you have comments and to let us know that you are using the forms.

COVID-19 CASE RECORD FORM RAPID version 08DEC2021

Adapted from SPRINT SARI CRF by ISARIC. Licensed under a Creative Commons Attribution-ShareAlike 4.0 International License by ISARIC on behalf of Oxford University.

PARTICIPANT ID I___I I___I I___I I___I I___I -- I___I I___I I___I I___I

MODULE1: complete on admission/enrolment

Site name _______________________ **Country** _______________________

Date of enrolment [D][D]/[M][M]/[2][0][Y][Y]

CLINICAL INCLUSION CRITERIA

Proven or suspected infection with pathogen of Public Health Interest ☐Yes ☐No

One or more	A history of self-reported feverishness or measured fever of ≥ 38ºC	☐Yes ☐No
of these	Cough	☐Yes ☐No
during this	Dyspnoea (shortness of breath) OR Tachypnoea*	☐Yes ☐No
illness	Clinical suspicion of ARI despite not meeting criteria above	☐Yes ☐No

** respiratory rate ≥50 breaths/min for <1 year; ≥40 for 1-4 years; ≥30 for 5-12 years; ≥20 for ≥13 years*

Is COVID-19 the reason for hospital admission?
☐Yes, COVID-19 is the reason for hospital admission
☐No, the patient is admitted to hospital for a reason other than COVID-19

DEMOGRAPHICS

Sex at Birth ☐Male ☐Female ☐Not specified **Date of birth** [_D_][_D_]/[_M_][_M_]/[_Y_][_Y_][_Y_][_Y_]

If date of birth is unknown, record: **Age** [___][___][___]years OR [___][___]months

Healthcare Worker? ☐Yes ☐No ☐Unknown **Laboratory Worker?** ☐Yes ☐No ☐Unknown

Pregnant? ☐Yes ☐No ☐Unknown ☐N/A **If yes: Gestational weeks assessment** [___][___] weeks

PREVIOUS COVID-19 INFECTIONS

Has the patient had COVID-19 previously?
☐ No ☐ Yes - once previously ☐ Yes - twice previously ☐ Yes - three times previously
(*there is more space on the eCRF to capture this)*

First COVID-19 infection:
When did their first COVID infection occur? (MM/YYY) ___________
Was their first COVID infection confirmed by testing:
☐Yes, confirmed by testing ☐No, not confirmed by testing
Were they admitted to hospital for their first infection of COVID? ☐Yes ☐No

Second COVID-19 infection:
When did their second COVID infection occur? (MM/YYY) ___________
Was their second COVID infection confirmed by testing:
☐Yes, confirmed by testing ☐No, not confirmed by testing
Were they admitted to hospital for their second infection of COVID? ☐Yes ☐No

If data on this patient was previously recorded in this study, record the Participant Identification Number (PIN) previously used in the section below

RE-ADMISSION AND PREVIOUS PIN

Was the patient admitted previously or transferred from any other facility during this illness episode?
☐YES-admitted previously to this facility ☐YES–transferred from other facility ☐NO ☐Unknown

Number of previous admissions for this infection: ______

Has this patient's data been previously collected under a different patient number?: ☐YES ☐NO ☐Unknown

If YES, Participant Identification Number (PIN): ________________

COVID-19 CASE RECORD FORM RAPID version 08DEC21

Adapted from SPRINT SARI CRF by ISARIC. Licensed under a Creative Commons Attribution-ShareAlike 4.0 International License by ISARIC on behalf of Oxford University.

World Health Organization ISARIC

PARTICIPANT ID I___I I___I I___I I___I I___I -- I___I I___I I___I I___I

MODULE1: complete on admission/enrolment

SYMPTOM ONSET AND ADMISSION *(first available data at presentation/admission)*

Symptom onset (date of first/earliest symptom) [_D_][_D_]/[_M_][_M_]/[_2_][_0_][_Y_][_Y_]

Admission date at this facility [_D_][_D_]/[_M_][_M_]/[_2_][_0_][_Y_][_Y_]

Temperature [___][___].[___]°C **Heart rate** [___][___][___]beats/min

Respiratory rate [___][___]breaths/min

BP [___] [___] [___](systolic) [___][___][___](diastolic) mmHg **Severe dehydration** ☐Yes ☐No ☐Unknown

Sternal capillary refill time >2seconds ☐Yes ☐No ☐Unknown

Oxygen saturation: [___][___][___]% on ☐room air ☐oxygen therapy ☐Unknown **A V P U** (circle one)

Glasgow Coma Score (GCS /15) [___][___] **Malnutrition** ☐Yes ☐No ☐Unknown

Mid-upper arm circumference [___][___][___]mm **Height:** [___] [___] [___]cm **Weight:** [___][___][___]kg

CO-MORBIDITIES *(existing prior to admission) (Unk = Unknown)*

Chronic cardiac disease *(not hypertension)*	☐Yes	☐No	☐Unk	Diabetes	☐Yes	☐No	☐Unk
Hypertension	☐Yes	☐No	☐Unk	Current smoking	☐Yes	☐No	☐Unk
Chronic pulmonary disease	☐Yes	☐No	☐Unk	Tuberculosis	☐Yes	☐No	☐Unk
Asthma	☐Yes	☐No	☐Unk	Asplenia	☐Yes	☐No	☐Unk
Chronic kidney disease	☐Yes	☐No	☐Unk	Malignant neoplasm	☐Yes	☐No	☐Unk
Chronic liver disease	☐Yes	☐No	☐Unk	Other	☐Yes	☐No	☐Unk
Chronic neurological disorder	☐Yes	☐No	☐Unk	If yes, specify:			
HIV	☐Yes-on ART	☐Yes-not on ART	☐No	☐Unknown			

PRE-ADMISSION & CHRONIC MEDICATION **Were any of the following taken within 14 days of admission?**

Angiotensin converting enzyme inhibitors (ACE inhibitors)? ☐Yes ☐No ☐Unknown

Angiotensin II receptor blockers (ARBs)? ☐Yes ☐No ☐Unknown

Non-steroidal anti-inflammatory (NSAID)? ☐Yes ☐No ☐Unknown

SIGNS AND SYMPTOMS ON ADMISSION *(Unk = Unknown)*

History of fever	☐Yes	☐No	☐Unk	Lower chest wall indrawing	☐Yes	☐No	☐Unk
Cough	☐Yes	☐No	☐Unk	Headache.	☐Yes	☐No	☐Unk
with sputum production	☐Yes	☐No	☐Unk	Altered consciousness/confusion	☐Yes	☐No	☐Unk
with haemoptysis	☐Yes	☐No	☐Unk	Seizures	☐Yes	☐No	☐Unk
Sore throat	☐Yes	☐No	☐Unk	Abdominal pain	☐Yes	☐No	☐Unk
Runny nose (rhinorrhoea).	☐Yes	☐No	☐Unk	Vomiting / Nausea	☐Yes	☐No	☐Unk
Wheezing	☐Yes	☐No	☐Unk	Diarrhoea	☐Yes	☐No	☐Unk
Chest pain.	☐Yes	☐No	☐Unk	Conjunctivitis	☐Yes	☐No	☐Unk
Muscle aches (myalgia)	☐Yes	☐No	☐Unk	Skin rash	☐Yes	☐No	☐Unk
Joint pain (arthralgia).	☐Yes	☐No	☐Unk	Skin ulcers	☐Yes	☐No	☐Unk
Fatigue / Malaise	☐Yes	☐No	☐Unk	Lymphadenopathy	☐Yes	☐No	☐Unk
Shortness of breath .	☐Yes	☐No	☐Unk	Bleeding (Haemorrhage).	☐Yes	☐No	☐Unk
Inability to walk	☐Yes	☐No	☐Unk	If bleeding: specify site(s):			

Other ☐Yes ☐No ☐Unk If yes, specify: ____________________

Adapted from SPRINT SARI CRF by ISARIC. Licensed under a Creative Commons Attribution-ShareAlike 4.0 International License by ISARIC on behalf of Oxford University.

World Health Organization ISARIC

PARTICIPANT ID I___I I___I I___I I___I I___I -- I___I I___I I___I I___I

MODULE1: complete on admission/enrolment

VACCINATIONS

Covid-19 vaccination ☐YES ☐NO ☐Unk

Date of first vaccine :[_D_][_D_]/[_M_][_M_]/[_2_][_0_][_Y_][_Y_] Date: ☐actual ☐estimated

Type of first vaccine: ☐Pfizer/BioNTech | ☐AstraZeneca/University of Oxford (Covishield in India) | ☐Moderna
☐Novavax | ☐Janssens (Johnson & Johnson) | ☐Sinopharm | ☐Sinovac | ☐Sputnik V
☐Covaxin | ☐CanSinoBIO | ☐Unknown | ☐other, please specify ____________________

Date of second vaccine :[_D_][_D_]/[_M_][_M_]/[_2_][_0_][_Y_][_Y_] Date: ☐actual ☐estimated

Type of second vaccine: ☐Pfizer/BioNTech | ☐AstraZeneca/University of Oxford (Covishield in India) | ☐Moderna
☐Novavax | ☐Janssens (Johnson & Johnson) | ☐Sinopharm | ☐Sinovac | ☐Sputnik V
☐Covaxin | ☐CanSinoBIO | ☐Unknown | ☐other, please specify ____________________

Date of third vaccine :[_D_][_D_]/[_M_][_M_]/[_2_][_0_][_Y_][_Y_] Date: ☐actual ☐estimated

Type of third vaccine: ☐Pfizer/BioNTech | ☐AstraZeneca/University of Oxford (Covishield in India) | ☐Moderna
☐Novavax | ☐Janssens (Johnson & Johnson) | ☐Sinopharm | ☐Sinovac | ☐Sputnik V
☐Covaxin | ☐CanSinoBIO | ☐Unknown | ☐other, please specify ____________________

Influenza vaccination within the last 6 months: ☐YES ☐NO ☐Unknown

Date of influenza vaccine :[_D_][_D_]/[_M_][_M_]/[_2_][_0_][_Y_][_Y_] Date: ☐actual ☐estimated

MEDICATION ***Is the patient CURRENTLY receiving any of the following?***

Oral/orogastric fluids? ☐Yes ☐No ☐ Unknown **Intravenous fluids?** ☐Yes ☐No ☐Unknown

Antiviral? ☐Yes ☐No ☐Unknown **If yes:** ORibavirin OLopinavir/Ritonavir ONeuraminidase inhibitor
OInterferon alpha OInterferon beta OOther, specify: ____________________

Corticosteroid? ☐Yes ☐No ☐Unknown **If yes,** route: OOral OIntravenous OInhaled

If yes, please provide agent and maximum daily dose: ____________________

Antibiotic? ☐Yes ☐No ☐Unknown **Antifungal agent?** ☐Yes ☐No ☐Unknown

Antimalarial agent? ☐Yes ☐No ☐Unknown **If yes,** specify: ____________________

Experimental agent? ☐Yes ☐No ☐Unknown **If yes,** specify: ____________________

Non-steroidal anti-inflammatory (NSAID) ☐Yes ☐No ☐Unknown

Angiotensin converting enzyme inhibitors (ACE inhibitors) ☐Yes ☐No ☐Unknown

Angiotensin II receptor blockers (ARBs) ☐Yes ☐No ☐Unknown

SUPPORTIVE CARE ***Is the patient CURRENTLY receiving any of the following?***

ICU or High Dependency Unit admission? ☐Yes ☐No ☐Unknown

Oxygen therapy? ☐Yes ☐No ☐ Unknown **If yes**, complete all below

O_2 flow: ☐1-5 L/min ☐6-10 L/min ☐11-15 L/min ☐>15 L/min ☐Unknown

Source of oxygen: ☐Piped ☐Cylinder ☐Concentrator ☐Unknown

Interface: ☐Nasal prongs ☐HF nasal cannula ☐Mask ☐Mask with reservoir ☐CPAP/NIV mask ☐Unknown

Non-invasive ventilation? *(e.g.BIPAP/CPAP)* ☐Yes ☐No ☐N/A

Invasive ventilation *(Any)*? ☐Yes ☐No ☐ Unknown **Inotropes/vasopressors?** ☐Yes ☐No ☐Unknown

Extracorporeal (ECMO) support? ☐Yes ☐No ☐ Unknown **Prone position?** ☐Yes ☐No ☐ Unknown

Adapted from SPRINT SARI CRF by ISARIC. Licensed under a Creative Commons Attribution-ShareAlike 4.0 International License by ISARIC on behalf of Oxford University.

World Health Organization ISARIC

PARTICIPANT ID I___I I___I I___I I___I I___I -- I___I I___I I___I I___I

MODULE1: complete on admission/enrolment

LABORATORY RESULTS ON ADMISSION *(*record units if different from those listed)*

Parameter	Value*	Not done	Parameter	Value*	Not done
Haemoglobin (g/L)		☐	Creatinine (μmol/L)		☐
WBC count (x10^9/L)		☐	Sodium (mEq/L)		☐
Haematocrit (%)		☐	Potassium (mEq/L)		☐
Platelets (x10^9/L)		☐	Procalcitonin (ng/mL)		☐
APTT/APTR		☐	CRP (mg/L)		☐
PT (seconds)		☐	LDH (U/L)		☐
INR		☐	Creatine kinase (U/L)		☐
ALT/SGPT (U/L)		☐	Troponin (ng/mL)		☐
Total bilirubin (μmol/L)		☐	ESR (mm/hr)		☐
AST/SGOT (U/L)		☐	D-dimer (mg/L)		☐
Urea (BUN) (mmol/L)		☐	Ferritin (ng/mL)		☐
Lactate (mmol/L)		☐	IL-6 (pg/mL)		☐

Adapted from SPRINT SARI CRF by ISARIC. Licensed under a Creative Commons Attribution-ShareAlike 4.0 International License by ISARIC on behalf of Oxford University.

World Health Organization ISARIC

PARTICIPANT ID I___I I___I I___I I___I I___I -- I___I I___I I___I I___I

MODULE 2: follow-up (frequency of completion determined by available resources)

Date of follow up [_D_][_D_]/[_M_][_M_]/[_2_][_0_][_Y_][_Y_]

VITAL SIGNS *(record most abnormal value between 00:00 to 24:00)*

Temperature [___][___].[___]°C **Heart rate** [___][___][___]beats per min **Respiratory rate** [___][___]breaths/min

BP [___] [___] [___](systolic) [___][___][___](diastolic) mmHg **Severe dehydration** ☐Yes ☐No ☐Unknown

Sternal capillary refill time >2seconds ☐Yes ☐No ☐Unknown **GCS/15** [___][___]

Oxygen saturation [___][___][___]% on ☐ room air ☐ oxygen therapy ☐Unknown **A V P U** (circle one)

DAILY CLINICAL FEATURES *(Unk = Unknown)*

Cough	☐Yes	☐No	☐Unk	Seizures	☐Yes	☐No	☐Unk
and sputum production	☐Yes	☐No	☐Unk	Vomiting / Nausea	☐Yes	☐No	☐Unk
Sore throat	☐Yes	☐No	☐Unk	Diarrhoea	☐Yes	☐No	☐Unk
Chest pain	☐Yes	☐No	☐Unk	Conjunctivitis	☐Yes	☐No	☐Unk
Shortness of breath	☐Yes	☐No	☐Unk	Myalgia	☐Yes	☐No	☐Unk
Confusion	☐Yes	☐No	☐Unk	Other, specify: ________	☐Yes	☐No	☐Unk

LABORATORY RESULTS *(*record units if different from those listed)*

Parameter	Value*	Not done	Parameter	Value*	Not done
Haemoglobin (g/L)		☐	Creatinine (μmol/L)		☐
WBC count (x10^9/L)		☐	Sodium (mEq/L)		☐
Haematocrit (%)		☐	Potassium (mEq/L)		☐
Platelets (x10^9/L)		☐	Procalcitonin (ng/mL)		☐
APTT/APTR		☐	CRP (mg/L)		☐
PT (seconds)		☐	LDH (U/L)		☐
INR		☐	Creatine kinase (U/L)		☐
ALT/SGPT (U/L)		☐	Troponin (ng/mL)		☐
Total bilirubin (μmol/L)		☐	ESR (mm/hr)		☐
AST/SGOT (U/L)		☐	D-dimer (mg/L)		☐
Urea (BUN) (mmol/L)		☐	Ferritin (ng/mL)		☐
Lactate (mmol/L)		☐	IL-6 (pg/mL)		☐

MEDICATION ***Is the patient CURRENTLY receiving any of the following?***

Oral/orogastric fluids? ☐Yes ☐No ☐ Unknown **Intravenous fluids?** ☐Yes ☐No ☐Unknown

Antiviral? ☐Yes ☐No ☐Unknown **If yes:** ORibavirin OLopinavir/Ritonavir ONeuraminidase inhibitor
OInterferon alpha OInterferon beta OOther, specify: ____________________

Corticosteroid? ☐Yes ☐No ☐Unknown **If yes,** route: OOral OIntravenous OInhaled

If yes, please provide agent and maximum daily dose: ____________________

Antibiotic? ☐Yes ☐No ☐Unknown **Antifungal agent?** ☐Yes ☐No ☐Unknown

Antimalarial agent? ☐Yes ☐No ☐Unknown **If yes,** specify: ____________________

Experimental agent? ☐Yes ☐No ☐Unknown **If yes,** specify: ____________________

Non-steroidal anti-inflammatory (NSAID) ☐Yes ☐No ☐Unknown

Angiotensin converting enzyme inhibitors (ACE inhibitors) ☐Yes ☐No ☐Unknown

Angiotensin II receptor blockers (ARBs) ☐Yes ☐No ☐Unknown

SUPPORTIVE CARE ***Is the patient CURRENTLY receiving any of the following?***

ICU or High Dependency Unit admission? ☐Yes ☐No ☐Unknown

Oxygen therapy? ☐Yes ☐No ☐Unknown **If yes,** complete all below:

O_2 flow volume: ☐1-5 L/min ☐6-10 L/min ☐11-15 L/min ☐>15 L/min ☐Unknown

Source of oxygen: ☐Piped ☐Cylinder ☐Concentrator ☐Unknown

Interface: ☐Nasal prongs ☐HF nasal cannula ☐Mask ☐Mask with reservoir ☐CPAP/NIV mask ☐Unknown

Non-invasive ventilation? *(e.g. BIPAP, CPAP)* ☐Yes ☐No ☐Unknown

Invasive ventilation *(Any)*? ☐Yes ☐No ☐Unknown **Inotropes/vasopressors?** ☐Yes ☐No ☐Unknown

Extracorporeal (ECMO) support? ☐Yes ☐No ☐Unknown **Prone position?** ☐Yes ☐No ☐ Unknown

Renal replacement therapy (RRT) or dialysis? ☐Yes ☐No ☐Unknown

Adapted from SPRINT SARI CRF by ISARIC. Licensed under a Creative Commons Attribution-ShareAlike 4.0 International License by ISARIC on behalf of Oxford University.

MODULE 3: complete at discharge/death

DIAGNOSTIC/PATHOGEN TESTING

Chest X-Ray /CT performed? ☐Yes ☐No ☐Unknown **If Yes: infiltrates present?** ☐Yes ☐No ☐Unknown
Was pathogen testing done during this illness episode? ☐Yes ☐No ☐Unknown **If yes**, complete all below:
Influenza virus: ☐Positive ☐Negative ☐Not done **If positive,** type ______________
Coronavirus: ☐Positive ☐Negative ☐Not done **If positive:** ☐MERS-CoV ☐SARS-CoV-2 ☐Other _________
Other respiratory pathogen: ☐Positive ☐Negative ☐Not done **If positive,** specify ______________________
Viral haemorrhagic fever: ☐Positive ☐Negative ☐Not done **If positive,** specify virus ___________________
Other pathogen of public health interest detected: If yes, specify: ________________________
Falciparum malaria: ☐Positive ☐Negative ☐Not done **Non-falciparum malaria:** ☐Positive ☐Negative ☐Not done
HIV: ☐Positive ☐Negative ☐Not done

COMPLICATIONS: At any time during hospitalisation did the patient experience:

Shock	☐Yes ☐No ☐Unknown	Bacteraemia	☐Yes ☐No ☐Unknown
Seizure	☐Yes ☐No ☐Unknown	Bleeding	☐Yes ☐No ☐Unknown
Meningitis/Encephalitis	☐Yes ☐No ☐Unknown	Endocarditis	☐Yes ☐No ☐Unknown
Anaemia	☐Yes ☐No ☐Unknown	Myocarditis/Pericarditis	☐Yes ☐No ☐Unknown
Cardiac arrhythmia	☐Yes ☐No ☐Unknown	Acute renal injury	☐Yes ☐No ☐Unknown
Cardiac arrest	☐Yes ☐No ☐Unknown	Pancreatitis	☐Yes ☐No ☐Unknown
Pneumonia	☐Yes ☐No ☐Unknown	Liver dysfunction	☐Yes ☐No ☐Unknown
Bronchiolitis	☐Yes ☐No ☐Unknown	Cardiomyopathy	☐Yes ☐No ☐Unknown
Acute Respiratory Distress Syndrome	☐Yes ☐No ☐Unknown	Other<br>If Yes, specify	☐Yes ☐No ☐Unknown<br>______________

MEDICATION: While hospitalised or at discharge, were any of the following administered?

Oral/orogastric fluids? ☐Yes ☐No ☐Unknown **Intravenous fluids?** ☐Yes ☐No ☐Unknown
Antiviral? ☐Yes ☐No ☐Unknown **If yes:** **O**Ribavirin **O**Lopinavir/Ritonavir **O**Neuraminidase inhibitor
OInterferon alpha **O**Interferon beta **O**Other, specify: ______________________________
Antibiotic? ☐Yes ☐No ☐Unknown **If yes,** specify: ________________
Corticosteroid? ☐Yes ☐No ☐Unknown **If yes,** route: **O**Oral **O**Intravenous **O**Inhaled
If yes, specify agent and maximum daily dose: ____________________
Antifungal agent? ☐Yes ☐No ☐Unknown **If yes,** specify: ________________
Antimalarial agent? ☐Yes ☐No ☐Unknown **If yes,** specify: ________________
Experimental agent? ☐Yes ☐No ☐Unknown **If yes,** specify: ________________
Non-steroidal anti-inflammatory (NSAID) ☐Yes ☐No ☐Unknown **If yes,** specify: __________________

SUPPORTIVE CARE: At ANY time during hospitalisation, did the patient receive/undergo:

ICU or High Dependency Unit admission? ☐Yes ☐No ☐ Unknown **If yes,** total duration: _________days
Date of ICU admission:[_D_][_D_]/[_M_][_M_]/[_2_][_0_][_Y_][_Y_] ☐N/A
Date of ICU discharge:[_D_][_D_]/[_M_][_M_]/[_2_][_0_][_Y_][_Y_] ☐in ICU at outcome ☐N/A
Oxygen therapy? ☐Yes ☐No ☐Unknown **If yes, complete all:** **Total duration:** _________days
O_2 flow volume: **O**1-5 L/min **O**6-10 L/min **O**11-15 L/min **O**>15 L/min
Source of oxygen: **O**Piped **O**Cylinder **O**Concentrator
Interface: **O**Nasal prongs **O**HF nasal cannula **O**Mask **O**Mask with reservoir **O**CPAP/NIV mask
Non-invasive ventilation? *(e.g. BIPAP, CPAP)* ☐Yes ☐No ☐ Unknown **If yes,** total duration: _________days
Invasive ventilation *(Any)*? ☐Yes ☐No ☐Unknown **If yes,** total duration: _________days
Extracorporeal (ECMO) support? ☐Yes ☐No ☐Unknown **If yes,** total duration: _________days
Prone position? ☐Yes ☐No ☐ Unknown **If yes,** total duration: _________days
Renal replacement therapy (RRT) or dialysis? ☐Yes ☐No ☐Unknown
Inotropes/vasopressors? ☐Yes ☐No ☐Unknown **If yes,** total duration: _________days

Adapted from SPRINT SARI CRF by ISARIC. Licensed under a Creative Commons Attribution-ShareAlike 4.0 International License by ISARIC on behalf of Oxford University.

MODULE 3: complete at discharge/death

OUTCOME

Is the patient infected with a variant of concern (VOC) ?

☐ Unknown
☐ No: Variant is known and no VOC identified
☐ Yes: Delta - B.1.617.2, identified Oct 2020
☐ Yes: Omicron, B.1.1.529, identified Nov 2021
☐ Yes: Alpha - B.1.1.7, identified in UK Sept 2020
☐ Yes: Beta - B.1.351, identified in South Africa May 2020
☐ Yes: Gamma - P.1, identified in Brazil Nov 2020
☐ Yes: Epsilon - B.1.427/B.1.429, identified in USA Mar 2021
☐ Yes: Zeta - P.2, identified in Brazil Apr 2020
☐ Yes: Eta - B.1.525, identified in Multiple Countries Dec 2020
☐ Yes: Theta - P.3, identified in Philippines Jan 2021
☐ Yes: Iota - B.1.526, identified in USA Nov 2020
☐ Yes: Kappa - B.1.617.1, identified in India Oct 2020
☐ Yes: Lambda - C.37, identified in Peru Dec 2020
☐ Yes: Mu - B.1.621, identified in Colombia Jan 2021
☐ Yes: A variant not listed above

Please check the REDCAP database for variants not listed above. New variants will be added to the database as they are identified.

If omicron variant was identified, what method was used to identify it?

☐Genomic sequencing ☐S-gene target failure (SGTF) testing ☐PCR genotyping ☐Unknown or untested

Outcome: ☐Discharged alive ☐Hospitalized ☐Transfer to other facility ☐Death ☐Palliative discharge ☐Unknown

Outcome date: [_D_][_D_]/[_M_][_M_]/[_2_][_0_][_Y_][_Y_] ☐Unknown

If Discharged alive: Ability to self-care at discharge versus before illness: ☐Same as before illness ☐Worse ☐Better ☐Unknown

Adapted from SPRINT SARI CRF by ISARIC. Licensed under a Creative Commons Attribution-ShareAlike 4.0 International License by ISARIC on behalf of Oxford University.
